# Demography, common disorders and mortality of Boxer dogs under primary veterinary care in the UK

**DOI:** 10.1186/s40575-023-00129-w

**Published:** 2023-06-01

**Authors:** Dan G. O’Neill, Alison M. Skipper, Kate Barrett, David B. Church, Rowena M. A. Packer, Dave C. Brodbelt

**Affiliations:** 1 Pathobiology and Population Sciences, The Royal Veterinary College, Hawkshead Lane, North Mymms, Hatfield, AL9 7TA Herts UK; 2 Clinical Science and Services, The Royal Veterinary College, Hawkshead Lane, North Mymms, Hatfield, AL9 7TA Herts UK

**Keywords:** VetCompass, Electronic patient record, EPR, Breed, Dog, Epidemiology, Primary-care, Veterinary, Pedigree, Purebred

## Abstract

**Background:**

The Boxer is a popular dog breed with a distinctive appearance. However, the breed has been linked with several health conditions, some of which have been associated with its moderately brachycephalic conformation and its white colouration. Anonymised primary-care veterinary clinical records were explored to extract data on the demography, common disorders and mortality of Boxers in the UK in 2016.

**Results:**

The study population of 336,865 dogs included 3,219 (0.96%) Boxers, of which 10.71% were recorded as white. The mean adult bodyweight was 30.43 kg (SD 5.73 kg). Annual disorder counts did not differ statistically between the sexes or between white and non-white Boxers. The most prevalent fine-level precision disorders were otitis externa (*n* = 230, 7.15%), epulis (188, 5.84%), corneal ulceration (161, 5.00%) and periodontal disease (149, 4.63%). Of the 34 most common fine-level disorders, none differed in prevalence between white and non-white dogs. The most prevalent disorder groups were skin disorder (*n* = 571, 17.74%), neoplasia (457, 14.20%) and ear disorder (335, 10.41%). White Boxers had higher prevalence than non-white Boxers for two disorder groups: dental disorder and brain disorder. The median longevity of 346 Boxers that died during the study was 10.46 years (IQR 9.00–11.98, range 2.76–18.00). Median longevity did not differ statistically between the sexes or between white and non-white Boxers. The most common grouped causes of death were death – unrecorded cause (*n* = 73, 21.10%), neoplasia (43, 12.43%) and brain disorder (33, 9.54%).

**Conclusions:**

There was minimal evidence of substantial health differences between white and non-white Boxers. Among the four most common disorders recorded in Boxers, two were typically common across all types of dogs (otitis externa and periodontal disease) while two suggested strong predispositions for the Boxer breed (epulis and corneal ulceration), showing the value of eliciting breed-specific disorder patterns for insights for potential health reforms. The overall longevity of Boxer dogs was consistent with other breeds of similar body size.

## Abstract (plain language summary)

The Boxer is a medium-large sized, active dog with a short coat and a moderately flat-faced (brachycephalic) skull shape. Despite many decades of popularity in the UK, there is limited published evidence on the overall health profile of Boxers. White-coloured Boxers are thought to be at higher risk of deafness from birth and as a result, white Boxers have traditionally often been culled at birth by breeders.

Using anonymised veterinary clinical records collected within the VetCompass Programme at the Royal Veterinary College (RVC), Boxers made up around 1% of all dogs in the UK in 2016, with an average adult bodyweight around 30 kg. The most common colours were brindle (22.82%), dark red (22.62%) and dark red multi-colour (20.92%), with 10.71% white Boxers. Across the 3,219 Boxers in the study, 73.97% had at least one disorder recorded during 2016. The average number of disorders per year did not differ statistically between female and male Boxers, or between white and non-white Boxers. The most common specific disorders in Boxers were ear infection (7.15%), gum mass (5.84%), eye ulcer (5.00%) and dental disease (4.63%). White and non-white dogs did not differ in their risk for any of the 34 most common specific disorders. Only two dogs in the study were recorded with deafness; one white and one non-white. The average lifespan of Boxers overall was 10.46 years. Lifespan did not differ statistically between female and male Boxers, or between white and non-white Boxers.

This study identifies ongoing popularity for the Boxer in the UK, with around 10% of these Boxers being white. There was minimal evidence of health differences between male and female Boxers, or between white and non-white Boxers. Among the four most common disorders recorded in Boxers, two are typically common across all types of dogs (ear and dental disease) while two showed strong predisposition in the Boxer breed (gum mass and eye ulcer), suggesting the value of understanding breed-specific health patterns to prioritise health plans for each breed. The overall longevity of Boxer dogs was typical of other breeds of this body size.

## Background

The Boxer was developed in late nineteenth century Germany, through the deliberate cross-breeding of a traditional large hunting breed, the Bullenbeisser, with English Bulldogs and other breeds, to produce an athletic working dog with a stable temperament [1]. The breed was brought to the U.K. during the 1930s, and first registered with The Kennel Club (UK) in 1939 [2]. After the Second World War, the breed’s popularity in the UK rapidly increased, peaking at over 7,000 annual registrations in 1959, when they were the fourth most popular breed (approximately 6% of all dogs registered that year with The Kennel Club). Their numbers then roughly halved during the following two decades, before climbing to a second peak of over 10,000 annual registrations in 1997 (the eighth most popular breed in the UK; 4% of total registrations). In recent years, the breed has averaged 3–4,000 annual UK registrations and was the fourteenth most popular breed in the UK in 2020 [3].

The Boxer is a medium-large sized, active dog with a short coat and a moderately brachycephalic skull shape [4]. The Kennel Club (UK) categorises the breed in the working group, and currently classifies it in Group 2 on its Breed Watch scheme, citing ‘pinched nostrils’ as a point of possible concern to be monitored by judges in the show ring [5]. The breed standard calls for a muzzle one-third the total length of the head, with a broad upper jaw and an undershot lower jaw (mandibular prognathism). The Boxer’s limbs are long and straight. The breed standard colours are fawn or brindle, with or without limited white markings that should not exceed one-third of the ‘ground’ colour [4]. However, some Boxers are completely or mostly white, and these individuals, like genetically similar white dogs in other breeds such as Bull Terriers, are thought to be at higher risk of congenital sensorineural deafness, although little research has specifically investigated the incidence of deafness in Boxers [6, 7].

Over the last sixty years, several breed-related Boxer health problems have been reported in the Anglophone literature. The first epidemiological study of disease in UK pedigree dogs was carried out in 1962 [8]. In that study, Boxers were the second most common breed reported with ectropion, the seventh most common breed reported with entropion, the third most common breed reported with ‘prolonged soft palate’, and the seventh most common breed reported with skin fold dermatitis. However, these figures were not definitive for breed predispositions for these conformational issues, given the high popularity of the breed at that time and the basic methodology of this pioneering survey. In 1964, a leading human dentist alerted the veterinary profession to a ‘disastrous’ incidence of dental malocclusion in Boxers and other brachycephalic breeds, as a conformational issue which he attributed to selective breeding for a shorter face [9].

In 1980, progressive axonopathy, a novel autosomal recessive neurological disease, was described in Boxers in the UK [10]. Over the following decade, this was eliminated from the breed through collaborative selective breeding, and is no longer considered a problem in the UK Boxer population today [11]. This successful intervention has left a relatively proactive and transparent cultural legacy within the Boxer breed community, which maintains an active web page dedicated to tracking investigations into certain breed health problems [11]. The first high-quality full genome sequence of the domestic dog was published in 2005 and used a female Boxer dog called Tasha [12]. This specific animal was selected for sequencing because it had the lowest heterozygosity at a small set of loci suggesting high levels of inbreeding that would make the sequencing easier. However, the researchers also chose the Boxer breed for their sequencing because of their belief that a predisposition to cancers in Boxers would enable them to combine information from the canine and human genome sequences to improve their searches for genetic contributors to cancer in man [13].

In recent years, The Kennel Club, together with breed community representatives, has produced a Breed Health and Conservation Plan for Boxers, which includes a review of the recent literature on breed predispositions to disease. It collates the results of breed health surveys and other sources of information to provide an evidence-based overview of currently evidenced major health concerns in Boxers. This document lists cancer, cardiac disease (particularly aortic stenosis and arrhythmogenic right ventricular cardiomyopathy (ARVC)), juvenile kidney disease and skin disease as current key health priorities for the Boxer [14].

Previous epidemiological reports by The Kennel Club [14], the Boxer Breed Council Health Committee [11] and the insurers Agria (U.K. and Sweden) [15] have provided insight into the incidence of various health problems in Boxers. In addition, a review of the wider literature in 2018 identified evidence of some predisposition to 86 disorders in Boxer dogs [16]. The breed has also featured in previous VetCompass publications, which have, for example, demonstrated a high Boxer breed predisposition for hypothyroidism [17] and corneal ulceration [18]. However, no single previous study has specifically investigated the health of Boxers across the full range of disorders using data harvested from primary-care veterinary clinics rather than owner reports (where the data may be distorted through information or social desirability bias) or insurance data (which are biased towards disorders that are not excluded from insurance cover) [19].

Using veterinary clinical data from the VetCompass Programme [20], this study aimed to characterise the demography, common disorders and mortality of the general population of Boxers under primary veterinary care in the UK, with a special comparative focus between males and females. Focus was also placed on exploring potential health effects for white Boxers. The results from the current study could provide a reliable framework to assist reforms in breeding practices and ultimately contribute to improved health and welfare of Boxers.

## Materials and methods

The study population included all dogs under primary veterinary care at clinics participating in the VetCompass™ Programme during 2016. Dogs under veterinary care were defined as those with either a) at least one electronic patient record (EPR) (free-text clinical note, treatment or bodyweight) recorded during 2016 or b) at least one EPR recorded both before and after 2016. The VetCompass Programme collates de-identified EPR data from primary-care veterinary practices in the UK for epidemiological research [20]. Data fields available for VetCompass researchers include a unique animal identifier along with species, breed, date of birth, colour, sex, neuter status and bodyweight, and clinical information from free-form text clinical notes and treatments with relevant dates.

A cohort study design with a cross-sectional analysis of disorder data on dogs registered at participating practices was used to estimate the one-year period prevalence of the most commonly diagnosed disorders. Sample size calculations estimated that 2,903 dogs were needed to report the prevalence for a disorder having a frequency of 2.0% to a precision of 0.5% [21]. Ethics approval was obtained from the RVC Ethics and Welfare Committee (reference number 2015/1369).

Dogs recorded as Boxer breed were categorised as Boxer and all remaining dogs were categorised as non-Boxer. *Adult Bodyweight* (Kg) described the mean bodyweight recorded from all bodyweight data for dogs aged over 18 months. *Neuter* described the status of the dog (entire or neutered) at the final available EPR. *Age* described the age at December 31^st^ 2016, the final date by which dogs were categorised as cases or non-cases for each disorder.

The list of unique Boxer animal identification numbers was randomly ordered and the clinical records of all animals were reviewed manually in detail to extract the most definitive diagnoses recorded for all disorders recorded in the clinical records as existing during 2016. Disorders were recorded to an ontology extended from the VeNom Code list as previously described [22]. Elective (e.g. neutering) or prophylactic (e.g. vaccination) clinical events were not included. No distinction was made between pre-existing and incident disorder presentations. Disorders described within the clinical notes using presenting sign terms (e.g. ‘vomiting’ or 'vomiting and diarrhoea'), but without a formally recorded clinical diagnostic term, were included using the first clinical sign listed (e.g. vomiting). Mortality data (recorded cause, date and method of death) were extracted on all deaths at any date during the available EPR data.

The extracted diagnosis terms were mapped to a dual hierarchy of precision for analysis: fine-level precision and grouped-level precision as previously described [22]. Briefly, fine-level precision terms described the original extracted terms at the maximal diagnostic precision recorded within the clinical notes (e.g. *inflammatory bowel disease* would remain as *inflammatory bowel disease*). Grouped-level precision terms mapped the original diagnosis terms to a general level of diagnostic precision (e.g. *inflammatory bowel disease* would map to *enteropathy*).

Following data checking for internal validity and cleaning in Excel (Microsoft Office Excel 2016, Microsoft Corp.), analyses were conducted using Stata Version 16 (Stata Corporation). The sex, neuter status, colour, age and adult bodyweight for Boxers under veterinary care during 2016 were described. Continuous variables that were normally distributed were described using mean (standard deviation [SD]); otherwise the median (interquartile range [IQR] and range) was reported [23]. One-year (2016) period prevalence values were reported for common disorders along with 95% confidence intervals (CI) that described the probability of diagnosis at least once during 2016. Binomial CI were calculated using the Wilson method. The median age was reported for each common disorder. Prevalence values were reported overall and separately for males and females. The chi-square test was used to compare categorical variables and the Students t-test or Mann–Whitney U test to compare continuous variables as appropriate [23]. Statistical significance was set at the 5% level.

## Results

### Demography

The study population of 336,865 dogs from 438 clinics in the VetCompass database under veterinary care during 2016 included 3,219 (0.96%) Boxers. Twelve of these Boxers (0.37%) had unrecorded sex and neuter status. Of the Boxers with information available, 1,542 (48.08%) were female and 1,435 (44.75%) were neutered overall. The probability of neutering did not differ between the sexes (female 44.75% vs male 44.74%, *P* = 0.999). Adult bodyweight data were available for 2306/3219 dogs (71.64%). The mean adult bodyweight overall was 30.43 kg (SD 5.73 kg). The mean adult bodyweight of males (32.99 kg, SD 5.42 kg) was heavier than for females (27.56 kg, SD 4.57 kg) (*P* < 0.001). Age data were available for 3156/3219 dogs (98.04%). The median age of Boxers overall was 5.81 years (IQR 3.10–8.79, range 0.18–18.37). The distribution of ages (years) did not differ statistically between females (median 5.92, IQR 3.23–5.92, range 0.10–18.37) and males (median 5.72, IQR 2.96–8.68, range 0.38–16.21) (*P* = 0.071). Colour information was recorded for 2940/3219 (91.33%) dogs. Of these, 1,821 (61,94%) were recorded as solid colour and 1,119 (38.06%) were recorded as multi-colour. The most common colours overall were brindle (*n* = 671, 22.82%), dark red (665, 22.62%) and dark red multi-colour (615, 20.92%). There were 315 (10.71%) Boxers recorded as white (Table 1).

**Table 1:** Colour of Boxers under primary veterinary care at practices participating in the VetCompass™ Programme in the UK from January 1^st^, 2016 to December 31^st^, 2016 (*n* = 2,940)

Colour^a^	No	%
Brindle	671	22.82
Dark red	665	22.62
Dark red multicolour	615	20.92
Brindle multicolour	332	11.29
White	315	10.71
Light fawn	157	5.34
Light fawn multicolour	145	4.93
Black multicoloured	17	0.58
Black	13	0.44
Tricolour	8	0.27
Blue multicoloured	2	0.07
Total	2,940	100

^a^Count covers dogs with available data

### Disorder prevalence

Manual examination of the EPRs of all 3,219 Boxers yielded 5,022 unique disorder events recorded during 2016. There were 2,381 (73.97%) Boxers with at least one disorder recorded during 2016 while the remaining 26.03% had no disorder recorded and either presented for prophylactic management only or did not present at all during 2016. The median annual disorder count per Boxer during 2016 was 1 disorder (IQR 0–2, range 0–12). Annual disorder counts did not differ statistically between female (median 1, IQR 0–2, range 0–12) and male Boxers (median 1, IQR 1–2, range 0–9) (*P* = 0.654). Annual disorder counts did not differ statistically between white (median 1, IQR 1–2, range 0–8) and non-white Boxers (median 1, IQR 0–2, range 0–12) (*P* = 0.414).

There were 420 distinct fine-level disorders across the study dogs. The most prevalent fine-level precision disorders recorded were otitis externa (*n* = 230, 7.15%), epulis (188, 5.84%), corneal ulceration (161, 5.00%), periodontal disease (149, 4.63%), heart murmur (138, 4.29%) and skin mass (138, 4.29%). The median age for dogs with individual fine-level disorders varied from 2.70 years (thin) to 10.75 years (death—unknown cause). Among the 34 most common fine-level disorders, females were more likely to be diagnosed with three disorders (periodontal disease, skin mass, urinary incontinence) while males were more likely to be diagnosed with three disorders (heart murmur, aggression, aural discharge). Among the 34 most common fine-level disorders, there were no disorders with differing prevalence between white and non-white dogs (Table 2). Only two dogs were recorded with deafness during the year of the study; one was white and one was non-white.

**Table 2:** Prevalence of the most common disorders at a *fine-level of diagnostic precision* recorded in Boxers (*n* = 3,219) under primary veterinary care at UK practices participating in the VetCompass™ Programme from January 1^st^, 2016 to December 31^st^, 2016. Disorders with 40 or more events included

Fine-level Disorder	No	Overall prevalence %	95% CI^a^	Median age (years)	Female prevalence %	Male prevalence %	Sex *P*-value*	White prevalence	Non-white prevalence	Colour *P*-value**
Otitis externa	230	7.15	6.31–8.09	5.50	7.00	7.27	0.772	7.30	7.35	0.974
Epulis	188	5.84	5.08–6.70	8.75	6.36	5.35	0.223	5.71	5.83	0.935
Corneal ulceration	161	5.00	4.30–5.81	8.77	4.60	5.35	0.336	6.03	5.14	0.503
Periodontal disease	149	4.63	3.96–5.41	8.66	5.58	3.72	**0.012**	6.98	4.53	0.054
Heart murmur	138	4.29	3.64–5.04	7.55	3.50	4.98	**0.038**	4.13	4.38	0.835
Skin mass	138	4.29	3.64–5.04	8.70	5.32	3.36	**0.006**	4.76	4.38	0.756
Obesity	123	3.82	3.21–4.54	6.64	4.09	3.54	0.423	3.17	3.81	0.575
Overgrown nail(s)	118	3.67	3.07–4.37	6.77	4.02	3.24	0.239	3.17	3.81	0.575
Hypersensitivity allergy disorder	95	2.95	2.42–3.59	4.71	3.11	2.82	0.628	2.86	3.09	0.824
Aggression	90	2.80	2.28–3.42	5.96	1.56	3.96	** < 0.001**	2.54	2.93	0.694
Diarrhoea	85	2.64	2.14–3.25	2.87	2.33	2.94	0.284	2.86	2.67	0.843
Osteoarthritis	82	2.55	2.06–3.15	9.97	2.66	2.46	0.725	3.49	2.55	0.327
Post-operative complication	68	2.11	1.67–2.67	3.61	1.88	2.28	0.427	1.27	2.21	0.273
Urinary incontinence	65	2.02	1.59–2.57	9.91	3.70	0.48	** < 0.001**	1.90	2.02	0.891
Aural discharge	64	1.99	1.56–2.53	4.14	1.49	2.46	**0.049**	2.54	2.06	0.573
Claw injury	63	1.96	1.53–2.50	4.82	1.95	1.92	0.961	1.27	1.98	0.380
Mast cell tumour	60	1.86	1.45–2.39	7.24	2.01	1.74	0.575	2.54	1.90	0.444
Alopecia	59	1.83	1.42–2.36	6.21	2.14	1.56	0.223	1.90	1.87	0.962
Pyoderma	59	1.83	1.42–2.36	5.27	1.75	1.92	0.719	2.86	1.79	0.191
Lameness	57	1.77	1.37–2.29	5.87	1.56	1.98	0.362	2.54	1.71	0.298
Hypothyroidism	54	1.68	1.29–2.18	8.84	1.56	1.80	0.589	0.63	1.83	0.122
Thin	53	1.65	1.26–2.15	2.70	1.62	1.68	0.893	2.86	1.52	0.081
Conjunctivitis	52	1.62	1.23–2.11	4.98	1.56	1.68	0.779	2.22	1.60	0.415
Skin disorder	50	1.55	1.18–2.04	5.47	1.75	1.38	0.399	1.59	1.64	0.946
Limb mass	49	1.52	1.15–2.01	8.14	1.49	1.56	0.872	1.27	1.64	0.622
Death—unknown cause	47	1.46	1.10–1.94	10.75	1.49	1.38	0.793	2.54	1.26	0.067
Infectious canine tracheobronchitis	47	1.46	1.10–1.94	4.35	1.17	1.74	0.176	0.95	1.45	0.479
Seizure disorder	47	1.46	1.10–1.94	9.59	1.43	1.50	0.860	2.22	1.41	0.262
Vomiting	47	1.46	1.10–1.94	4.34	1.49	1.44	0.906	2.22	1.49	0.320
Anal sac impaction	46	1.43	1.07–1.90	5.51	1.10	1.74	0.128	0.95	1.60	0.376
Cruciate disease	43	1.34	0.99–1.79	6.15	0.97	1.68	0.081	0.95	1.41	0.508
Atopic dermatitis	42	1.30	0.97–1.76	5.01	1.49	1.14	0.383	2.22	1.26	0.162
Acrochordon	41	1.27	0.94–1.72	7.42	1.04	1.04	0.303	1.59	1.26	0.624
Stiffness	41	1.27	0.94–1.72	10.40	1.23	1.32	0.822	1.27	1.22	0.938

^a^CI confidence interval. *The *P*-value reflects prevalence comparison between females and males. **The *P*-value reflects prevalence comparison between white and non-white Boxers

There were 59 distinct disorder groups recorded across the study dogs. The most prevalent disorder groups were skin disorder (*n* = 571, 17.74%), neoplasia (457, 14.20%), ear disorder (335, 10.41%), mass lesion (325, 10.10%) and ophthalmological disorder (320, 9.94%). The median age for dogs with individual disorder groups varied from 3.11 years (underweight) to 9.78 years (brain disorder). Among the 22 most common grouped disorders, females were more likely to be diagnosed with three disorders (mass lesion, dental disorder, urinary system disorder) while males were more likely to be diagnosed with one disorder (behavioural disorder). Among the 22 most common grouped disorders, white Boxers had higher prevalence than non-white Boxers for two disorders: dental disorder and brain disorder (Table 3).

**Table 3:** Prevalence of the most common disorders at a *grouped-level of diagnostic precision* recorded in Boxers (*n* = 3,219) under primary veterinary care at UK practices participating in the VetCompass™ Programme from January 1^st^, 2016 to December 31^st^, 2016. Disorders with 50 or more events included

Grouped-level Disorder^a^	No	Overall prevalence %	95% CI*	Median age (years)	Female prevalence %	Male prevalence %	Sex *P*-value**	White prevalence	Non-white prevalence	Colour *P*-value**
Skin disorder	571	17.74	16.46–19.10	5.33	17.77	17.78	0.995	17.14	18.48	0.563
Neoplasia	457	14.20	13.03–15.45	8.15	14.85	13.45	0.256	15.24	14.32	0.663
Ear disorder	335	10.41	9.40–11.51	5.34	9.60	11.17	0.145	10.79	10.78	0.995
Mass lesion	325	10.10	9.10–11.19	8.75	11.87	8.47	**0.001**	9.52	10.55	0.573
Ophthalmological disorder	320	9.94	8.95–11.02	8.37	9.66	10.15	0.645	10.48	10.29	0.916
Musculoskeletal disorder	286	8.88	7.95–9.92	8.24	8.11	9.67	0.121	10.16	8.91	0.467
Enteropathy	250	7.77	6.89–8.74	3.86	7.26	8.23	0.308	9.84	7.81	0.210
Heart disease	204	6.34	5.55–7.23	7.93	5.51	7.09	0.067	6.35	6.44	0.952
Nail disorder	195	6.06	5.28–6.94	5.92	6.29	5.71	0.486	5.08	6.21	0.428
Dental disorder	170	5.28	4.56–6.11	8.64	6.16	4.44	**0.030**	7.94	5.14	**0.039**
Urinary system disorder	131	4.07	3.44–4.81	8.92	6.23	2.10	** < 0.001**	4.13	4.15	0.983
Obesity	123	3.82	3.21–4.54	6.64	4.09	3.54	0.423	3.17	3.81	0.575
Traumatic injury	112	3.48	2.90–4.17	3.28	2.92	3.96	0.106	2.54	3.47	0.389
Complication associated with clinical care procedure	108	3.36	2.79–4.03	4.71	3.57	3.12	0.485	3.17	3.50	0.762
Behavioural disorder	106	3.29	2.73–3.97	5.96	2.08	4.44	< 0.001	2.86	3.43	0.595
Upper respiratory tract disorder	104	3.23	2.67–3.90	4.22	2.85	3.60	0.231	4.13	3.12	0.341
Female reproductive disorder	83	2.58	2.08–3.19	4.55	5.32	0.06	~	1.90	2.70	0.401
Brain disorder	82	2.55	2.06–3.15	9.78	2.20	2.82	0.265	5.08	2.25	**0.003**
Underweight	71	2.21	1.75–2.77	3.11	2.14	2.28	0.784	3.49	2.02	0.090
Anal sac disorder	66	2.05	1.61–2.60	5.51	2.08	2.04	0.947	1.59	2.29	0.426
Endocrine system disorder	66	2.05	1.61–2.60	8.95	2.01	2.10	0.855	0.63	2.25	0.058
Male reproductive system disorder	56	1.74	1.34–2.25	4.65	~	3.36	~	2.86	1.68	0.137

^a^CI confidence interval. *The *P*-value reflects prevalence comparison between females and males. **The *P*-value reflects prevalence comparison between white and non-white Boxers

### Mortality

There were 346 deaths recorded in Boxers during the study. Information on age at death was available for 335 of these deaths. The median longevity of Boxers overall was 10.46 years (IQR 9.00–11.98, range 2.76–18.00). The median longevity of females (10.41 years, IQR 9.16–11.86, range 1.34–18.00, *n* = 171) did not differ statistically to males (10.53 years, IQR 8.60–12.00, range 1.62–15.43, *n* = 164) (*P* = 0.920). Coat colour was recorded for 310 of the deaths. The median longevity of white Boxers (10.55 years, IQR 9.00–11.70, range 3.65–14.51, *n* = 43) did not differ statistically to non-white Boxers (10.38 years, IQR 8.88–11.94, range 1.34–15.46, *n* = 262) (*P* = 0.791). The median age at death for dogs with individual disorder groups varied from 3.11 years (thin) to 9.78 years (brain disorder). The method of death was recorded for 334 deaths. Of these, 311 (93.11%) deaths were by euthanasia while 23 (6.89%) deaths were unassisted. The probability of death by euthanasia did not differ statistically between females (92.90%) and males (93.29%) (*P* = 0.887). The probability of death by euthanasia did not differ statistically between white (90.70%) and non-white Boxers (93.77%) (*P* = 0.454). The most common causes of death described at a grouped-precision level were death – unrecorded cause (*n* = 73, 21.10%), neoplasia (43, 12.43%), brain disorder (33, 9.54%) and mass lesion (29, 8.38%). The probability of death did not differ statistically between females and males for any of the 12 most common grouped causes of death. White Boxers had a higher probability of death than non-white Boxers for two of the 12 most common grouped causes of death: brain disorder and enteropathy (Table 4).

**Table 4:** Common grouped causes of mortality in Boxers under primary-care veterinary at UK practices participating in the VetCompass™ Programme from January 1^st^, 2016 to December 31^st^, 2016 (*n* = 346). Disorders with 8 or more events included

Grouped-level disorder	No	Overall %	95% CI^a^	Median age at death (years)	% Female	% Male	*P*-Value*	White prevalence	Non-white prevalence	White *P*-value**
Death – unrecorded cause	73	21.10	17.13–25.70	10.58	23.73	17.86	0.180	20.45	20.90	0.947
Neoplasia	43	12.43	9.36–16.32	9.45	11.86	13.10	0.729	9.09	12.69	0.499
Brain disorder	33	9.54	6.87–13.09	11.01	9.04	10.12	0.733	18.18	7.84	**0.028**
Mass lesion	29	8.38	5.90–11.78	10.95	8.47	8.33	0.962	4.55	9.70	0.267
Collapsed	23	6.65	4.47–9.78	11.61	6.21	7.14	0.730	4.55	7.46	0.484
Heart disease	16	4.62	2.87–7.38	10.13	3.95	5.36	0.536	0.00	5.22	0.121
Poor quality of life	11	3.18	1.78–5.60	10.55	2.26	4.17	0.314	2.27	3.73	0.627
Renal disease	11	3.18	1.78–5.60	7.92	3.95	2.38	0.406	2.27	3.73	0.627
Spinal cord disorder	10	2.89	1.58–5.24	10.84	1.69	4.17	0.171	4.55	2.61	0.478
Musculoskeletal disorder	9	2.60	1.37–4.87	12.01	2.26	2.98	0.677	2.27	2.61	0.895
Behaviour disorder	8	2.31	1.18–4.50	7.00	1.69	2.98	0.429	0.00	2.24	0.316
Enteropathy	8	2.31	1.18–4.50	8.77	3.39	1.19	0.175	6.82	1.49	**0.027**

^a^CI confidence interval. *The *P*-value reflects prevalence comparison between females and males. **The *P*-value reflects prevalence comparison between white and non-white Boxers

## Discussion

This study of over 3,000 Boxers among the wider domestic dog population in the UK has demonstrated an ongoing popularity of the breed, comprising around 1% of dogs in the UK in 2016. The study also identifies the most common disorders recorded in the breed, showing that some of these, such as otitis externa and periodontal disease, occur at levels typical of dogs overall while others, such as epulis and corneal ulceration, are quite breed predisposed. There was only limited evidence for important health differences between the sexes and very little evidence supporting reduced health in white Boxers. With an overall median longevity of 10.46 years, the typical lifespans of Boxers appeared to be largely consistent with other breeds of this body size. This study highlights the value of Big Data resources such as VetCompass to provide breed health profiles that can support improved health plans for individual breeds.

### Demography

Boxers comprised just under 1% of the total canine population surveyed in this 2016 dataset. In the same year, Boxers comprised 1.6% of dogs registered at The Kennel Club [3]. This higher proportion among the registered pedigree dogs is expected, given that the wider canine population also includes designer crossbreeds, mixed breeds and other breeds of dog that are not registered with or recognised by The Kennel Club. There were no statistically significant demographic differences between males and females except for bodyweight. Mean bodyweight for males (32.99 kg) averaged almost 5.5 kg more than females (27.56 kg). This mirrors the sexual dimorphism encoded in the breed standard, which suggests a male bodyweight of 30–32 kg and a female bodyweight of 25–27 kg [4]. In both sexes, the mean bodyweights reported through VetCompass analysis exceeded the upper limit recommended by the breed standard, suggesting that either the wider general population is truly comprised of structurally larger dogs than the idealised view of the breed or that a population tendency to overweight/obesity is skewing the ideal bodyweight results.

Just over 10% of the Boxers in this study cohort were recorded as white. Although this colour is not accepted under the breed standards of The Kennel Club (UK) or the Fédération Cynologique Internationale (FCI) [4, 24], white animals have existed in the breed since its foundation [25]. Gene mapping has shown a shared genetic mutation for the white colour in Boxers and Bull Terriers, and white colouring has been linked to congenital deafness for over a century [26, 27]. For this reason, white Boxers have long been considered undesirable by breeders and historically were often culled [28]. Consequently, little reliable information has been available until now on the proportion of white dogs within the Boxer breed or on associations of white colour with deafness and other health problems in the wider population. In a study in the Netherlands in 1994–5, 16.9% of a cohort of 2,629 Boxer puppies were euthanized at birth because they were white [29], and it is likely that similar culling practices prevailed elsewhere at that time. However, changing attitudes towards such large-scale culling among pedigree dogs now mean that more white Boxers are sold as pets and fewer are euthanized as puppies. A more recent study in Italy reported that 17.5% of a Boxer population in Italy were reported by their owners as white, with none of these white dogs recorded as deaf [28]. In the current VetCompass study, 10.71% of Boxers were recorded as white in the UK. This lower UK proportion of white Boxers could reflect a genuine difference in allele frequency between different national dog populations or might indicate some ongoing level of neonatal culling of white dogs in the UK. For comparison, The Kennel Club registration data for all Boxers recorded as born between 2015 and 2018 identified that 6.8% (1,022 from a total of 15,124) were white [3]. Although these Kennel Club data refer to births during these years rather than to the adult population that was reported in the VetCompass data, this even lower figure could reflect ongoing reluctance by breeders to register non-standard white dogs, particularly if they perceive these puppies as deaf or likely to be deaf. An alternative explanation could be that genetic mutations for white dogs are genuinely more common in the wider non-Kennel Club population of Boxers in the UK. The current study also failed to show any predisposition for deafness in the white Boxers. This may reflect previous culling of fully deaf dogs to mainly leave white Boxers with at least unilateral hearing in the living population. An alternative explanation could be that some of these living white Boxers were indeed deaf but that neither the owner or veterinary teams were aware of their deafness, which can be challenging to identify, or that this information was not recorded in the clinical notes [30].

### Disorder prevalence

Neither males versus females, nor white versus non-white Boxers differed statistically in their annual disorder counts, suggesting little or no overall health differences between these categories of dog. The two most prevalent disorder groups both overall and in each sex were skin disorder (overall 17.74%) and neoplasia (overall 14.20%). This finding provides supporting evidence for the breed health priorities of neoplasia and skin disease previously determined by The Kennel Club’s Breed Health and Conservation Plan [14], with the finding of mass lesion in the current study (overall 10.10%) as another common disorder group that was likely to also include many cases of neoplasia that had not been worked up to full diagnoses. There is a long and deep literature worldwide on predispositions to a range of neoplasia in Boxer dogs based on data sources including referral veterinary records [31], post-mortem studies [32], histopathology sample analyses [33], cancer registries [34, 35] and mortality studies [36]. The current study provides further supporting evidence of cancer predisposition in Boxers that is based on primary-care veterinary data. At a disorder group level, 14.2% of the Boxers in the current study were diagnosed with neoplasia, which is substantially higher than results using the same data source for other similar sized breeds such as German Shepherd Dog 4.82% [37], Greyhound 5.5% [38] Labrador Retriever 7.4% [39] and Rottweiler 7.96% [40]. The evidence for a cancer predisposition in the current study also supports the choice of Boxer dogs as comparative oncology models for translational research to better understand the origins of cancer and translate these findings to novel therapies, in line with the thinking of the team that chose the Boxer for the first full genomic sequencing in dogs [13, 41].

Delving deeper into health differences between the sexes, there were differences detected for some disorders at both grouped and fine levels of analysis. For example, at the grouped level, females were almost three times more likely than males to be diagnosed with a urinary system disorder: a difference that reflects an even more marked sex difference at the fine level of analysis, where females were almost eight times more likely than males to be diagnosed with urinary incontinence. This female predisposition for urinary incontinence in Boxers concurs with the relative risk of over 3 times for females compared to males that has previously been recorded in dogs overall in the UK [42, 43]. At the grouped disorder level, females were also significantly more likely than males to be diagnosed with mass lesion and dental disorder, while at the fine level of disorder, females were 1.5 times as likely as males to be diagnosed with skin mass and 1.5 times as likely to be diagnosed with periodontal disease. However, despite this sex predisposition, the prevalence of 5.58% for periodontal disease in females was still lower than the overall prevalence of 12.5% recorded across all dogs in a previous VetCompass study using the same methodology, suggesting that Boxers may be a relatively protected breed against periodontal disease overall [44]. This relative protection is also reflected in other brachycephalic breeds such as the French Bulldog and English Bulldog [45, 46]. Paradoxically, it is possible that the open mouthed breathing, dental malocclusion, mandibular prognathism, drooling and widely spaced teeth seen in many Boxers and other brachycephalic breeds, which have been considered problematic for over seventy years, are nevertheless somehow protective against gingivitis: perhaps by reducing food trapping and by increasing saliva flow [9].

At the grouped disorder level, males were over twice as likely (× 2.1) as females to be diagnosed with behavioural disorder. At the fine level of diagnostic precision, males were 2.5 times as likely to show aggression, which was recorded in 3.96% of males compared with 1.56% of females. Somewhat surprisingly, despite promotion by The Kennel Club as ‘much loved as a family companion for his intelligence and character’, aggression was the fifth most common fine-level disorder in male Boxers, reported more frequently than anecdotally ‘common’ disorders in primary care practice such as diarrhoea and osteoarthritis. Given that behavioural disorder was also the fourth commonest cause of mortality in this male cohort and showed the lowest median age of death, this suggests that behavioural problems, particularly aggression and especially in males, may have a significant impact on the welfare of Boxers and the people they live with, which potentially merits further attention [47]. This finding further corroborates reports of relatively high levels of aggression in Boxers compared to otherwise behaviourally similar dogs, identified via large-scale analysis of C-BARQ data on over 32,000 dogs in the USA [48]. However, although the 2.80% prevalence of aggression reported overall in Boxers in the current study was higher than the 1.09% aggression in Labrador Retriever [49] and 1,7% in Greyhounds [38] previously reported, it was substantially lower than the 4.76% aggression reported in German Shepherd Dogs [37] and 7.46% reported in Rottweilers [40]. This wide variation in the prevalence of aggression both between breeds, and also between sexes within breeds, suggests that improved information on behavioural and temperament attributes of individual breeds is needed to help to owner make appropriate decisions when selecting breeds as companion and family dogs.

At the fine level of disorders, males were also statistically more likely than females to be diagnosed with heart murmur, with a male prevalence of 4.98% compared to a female prevalence of 3.50%. Although heart murmur was the fifth most commonly recorded fine-level disorder overall, it jumped to the fourth most common fine-level disorder in males compared to only the ninth most common in females. While specific individual cardiological diagnoses, such as aortic stenosis and atrial septal defects, which can cause murmurs, have been recognised for many years as breed health concerns in Boxers, many true cases of these conditions in typical primary care caseloads may not be clinically worked up to this level of diagnostic precision [14]. Supporting the idea that clinical signs are often used in lieu of formal biomedical diagnoses in general veterinary care, previous work within VetCompass has reported that while just 0.36% of dogs under primary veterinary care were recorded with the specific diagnosis of degenerative mitral valve disease, there were almost nine times as many (3.18%) recorded and clinically managed for a health murmur diagnosis that was clinically consistent with the existence of degenerative mitral valve disease [50]. Nevertheless, the finding that heart murmurs are recorded more frequently in Boxer males is consistent with previous research that reported a male predisposition for both aortic and pulmonic stenosis in Boxers [51].

In addition to some disorder predispositions where the prevalence in Boxers is higher than in other types of dogs, the current study also highlights that the Boxer also has some disorders with concerningly high prevalence that are similarly common in dogs that are not Boxers. Although otitis externa was the most prevalent fine-level precision disorder in Boxers (7.15%), this prevalence was very similar to the 7.30% prevalence for otitis externa reported across all UK dogs in a previous VetCompass study that used a similar methodology [22]. None the less, it could be argued that the high frequency of otitis externa is suggestive that this disorder should be considered as a key health priority for the Boxer. There has been a tendency for many decades to focus breed health reforms on predispositions within breeds or on disorders where there is a genetic or other diagnostic test available, no matter how rare the actual disorders are, rather than on also considering common disorders that may not even have a predisposition [14]. It may be that veterinary and breeding communities need to give greater future emphasis in breed health plans to the sum of the health and welfare impacts at an overall breed level so that these health schemes truly deliver health and welfare gains at a population level.

However, that said, many of the most frequently reported fine-level disorders in the current study do show the double welfare impact of both high prevalence and high breed predisposition which marks these as very strong candidates for consideration as key disorder priorities for Boxers. Strong historical support for these disorders as breed-related problems in Boxers only further supports their prioritisation. Predisposition of Boxers to dental epulis (also known as peripheral odontogenic fibroma or fibromatous epulis of periodontal ligament origin) has been recognised since at least the 1960s [52]. More recently, this predisposition has been highlighted in a UK owner survey [53], in Swedish insurance data [14] and in UK diagnostic laboratory data [54]. In the current study, epulis was the second most prevalent fine-level disorder in both sexes, with an overall prevalence of 5.84%.

As the third most common disorder in Boxers in the current study, with a prevalence of 5.00%, corneal ulceration is another condition that is flagged by the current work with both high prevalence and predisposition. Conformational predisposition to corneal ulceration among dogs with brachycephaly has been recognised for over a century [55] with a breed-specific problem of non-healing (indolent) corneal ulceration formally described in Boxers in the 1960s [56]. Previous VetCompass work on corneal ulceration across multiple breeds identified the Boxer to have the second highest breed prevalence of corneal ulceration (4.98%) (exceeded only by the Pug at 5.42%), with the Boxer prevalence being 13 times higher than the 0.38% prevalence recorded in crossbreeds [18]. Such a high prevalence and predisposition for a painful condition that is linked to extreme body conformation suggests a very strong rationale for prioritising this condition within reforms that could include breeding towards conformational moderation to improve innate health in the breed [14, 57]. To build on this concept of evaluating disorder prioritisation within breeds based on both prevalence and predisposition, the current work also highlighted a relatively high prevalence for hypothyroidism, which affected 1.68% of the Boxer dogs in this study. Although this was only around one third of the prevalence for corneal ulceration, previous VetCompass work has reported the Boxer breed with the third highest prevalence of hypothyroidism and to have over 10 times the risk compared with crossbred dogs [17]. This comparison between corneal ulceration and hypothyroidism in Boxers highlights that even when a breed is highly predisposed to a certain disorder, a more prevalent disease leads to a higher disease burden and therefore may be considered as having a greater breed welfare impact. Other factors such as the duration and the severity of each disorder should also be considered with making decisions about breed health prioritisation [58].

There is growing awareness that identification of disorders that are protected against may also offer useful information for breed health plans. For best effect, studies that identify protections should include clinical data on both the breed of interest as well as on a wider comparator group e.g. all remaining dogs in the population that are not of this breed. To date, such comparative studies have been reported for breeds including French Bulldog [45], Pug [59], English Bulldog [46], Labrador Retriever [49] and Staffordshire Bull Terrier [60]. However, although the current study included only results on Boxer dogs, it is still possible to identify some evidence on disorder protections by comparing the current results to prevalence data on dogs overall that were derived from the same VetCompass data source [22]. By comparing the current results to the prevalence values for dogs overall in the UK, there was some evidence to suggest protection in Boxers to periodontal disease (prevalence 4.63% in Boxers vs 12.52% in dogs overall), obesity (3.82% vs 7.07%), overgrown nails (3.67% vs 5.52%) and diarrhoea (2.64% vs 3.81%). The existence of both protections as well as predispositions to differing disorders within breeds adds to the nuances needed to fully understand overall breed health and promotes the value of holistic approaches to promoting breed health such as are taken by the Kennel Club’s Breed Health and Conservation Plans [61].

Boxers are widely regarded as a breed with a moderately brachycephalic head shape [4]. However, current research increasingly draws nuanced distinctions between the typical attributes and pathologies from the brachycephalic conformation across different breeds in dogs [62-64]. Despite the strong predisposition in Boxers to corneal ulceration, which is typical for the brachycephalic conformation, it is important to consider whether every disorder predisposition linked to brachycephaly will also show predisposition in the Boxer. Brachycephalic airway obstructive syndrome (BOAS) is widely considered as a common disorder in dogs with extreme brachycephaly [65, 66]. A previous VetCompass study examining associations between brachycephaly and upper respiratory tract disorder across extreme, moderate and non-brachycephalic breeds of small and medium size reported a higher prevalence of upper respiratory tract disorders in extreme brachycephalic dogs (22%) than in the moderate and non-brachycephalic group (9.7%). It is therefore, somewhat surprising that the current Boxer study reveals an unexpectedly low grouped level prevalence for upper respiratory tract disorder of only 3.23%, suggesting that upper respiratory tract disorder does not carry a major welfare burden in Boxers and that perhaps its larger body size may be protective for the Boxer, despite its moderately brachycephalic features. The relatively higher craniofacial ratio (muzzle to overall skull length ratio) of the Boxer compared to more extreme brachycephalic breeds (Boxer: 0.31, vs. Pug: 0.08; French Bulldog: 0.19; English Bulldog: 0.22) has previously been reported to be associated with markedly lower predisposition to upper respiratory tract disorders in Boxers with a conclusion that the more moderate facial conformation of the Boxer likely results in reduced risk of anatomical abnormalities and subsequent overcrowding of the airways [67]. Other predispositions that are recorded in dogs overall with brachycephaly but that had lower relative prevalence in Boxers included umbilical hernia, pododermatitis, patellar luxation and anal sac impaction [64]. These results highlight the dangers of ecological fallacy whereby the disorder risks of a group (e.g. all breeds with brachycephaly) are attributed to an individual (e.g. the Boxer as a single breed with brachycephaly) and underlines the value of breed-specific epidemiological and clinical investigation such as the current study [68].

One aim of the current study was to investigate the health of white Boxers compared to non-white Boxers. The Kennel Club breed standard currently excludes white dogs, which have long been anecdotally considered to have an elevated risk of congenital sensorineural deafness and, possibly, other diseases [4, 28]. However, despite this, very little empirical research has been reported on the incidence of congenital sensorineural deafness in Boxers. One small study in Brazil carried out brainstem auditory-evoked response (BAER) testing on 43 Boxers, of which 14 were white, showing that two white dogs were bilaterally deaf and one was unilaterally deaf, while all the non-white dogs had normal hearing [7]. However, as those authors pointed out, the study sample size was too small to draw meaningful statistical conclusions. Another approach here is to extrapolate results from Bull Terriers, which have the same genetic cause for colour-related deafness as Boxers [6, 7], but have been studied in more detail, with these studies benefitting from a higher proportion of white dogs among Bull Terriers because white dogs are allowed within The Kennel Club breed standard [4]. A recent BAER study of 1060 Bull Terriers in the UK reported an overall 10.19% prevalence of congenital sensorineural deafness, with 8.21% unilaterally deaf and 1.98% bilaterally deaf [69]. When divided by colour, 19.29% of white dogs were deaf in one or both ears, whereas only 0.77% of the non-white dogs were deaf in one or both ears. Although these data refer to Bull Terriers, all other things being equal, a similar prevalence of deafness should be expected in white Boxers, given the common underlying cause. However, in the current VetCompass study, only two dogs (one white and one non-white) were identified as deaf, suggesting the potential for substantial under-reporting of deafness within primary care caseloads, perhaps because the owner and/or clinician were unaware of the problem, particularly in the case of unilateral deafness. Alternatively, the true proportion of deaf white dogs in the adult dog population may be low because culling as a default decision among breeders may still be common in the UK [28]. Overall, the current study identified only weak evidence for any significant difference in health between white and non-white Boxers. None of the 34 fine-level disorders showed prevalence differences between white and non-white Boxers while just two of the grouped-level disorders differed, with white Boxer having higher prevalence of brain disorders and enteropathy. Given the multiple testing of 66 individual tests across the fine-level and group disorders, it would not be unexpected for two tests to return false positive (type I) errors, so even these two findings should be treated with caution [70]. An absence of wider health differences between white and non-white Boxers apart from hearing issues is not surprising because, despite the long-standing prejudice against white colour in this breed, the causative mutation is not generally thought to be linked to health problems other than congenital deafness [71] and vulnerability to sunburn as in all animals with little pigmentation [72].

### Mortality

The median age at death for Boxers in the current study was 10.46 years, which is midway across the range for some other large-sized breeds previously reported using a similar methodology; Rottweiler (9.0 years) [40], German Shepherd Dogs (10.3 years) [37] and Labrador Retrievers (12.0 years) [39]. The longevity of Boxers reported here is shorter than the median of 12.0 years reported across all dogs in the UK but it is well recognised that smaller dogs have a longevity advantage over larger dogs [73]. Therefore, these results do not suggest that the longevity overall of Boxers is particularly shorter than might be expected for a pure breed of this body size. This is especially noteworthy given that there is strong evidence that extreme brachycephaly is linked to substantially shortened lifespan [74]. It may be that the moderate rather than extreme degree of brachycephaly alone of many Boxers does not impose meaningful lifespan reductions on this breed, which does not show extreme conformation in other parts of the body [4].

Among the common causes of mortality, four disorder groups were identified with markedly earlier ages at death than the overall mean age of 10.46 years described for this Boxer population: neoplasia (9.45 years); renal disease (7.92 years); behavioural disorder (7.00 years); and enteropathy (8.77 years). Given these early deaths, it could be considered that these disorders exert an important impact on Boxer health and welfare. It is noteworthy that of these, neoplasia and renal disease map onto two priority health concerns reported by the breed community, cancer and juvenile kidney disease [14]. Given the strong prior evidence base on predisposition to neoplasia in Boxers [32, 36, 75], the current findings of high rates of mortality from neoplasia are not surprising. However, even these may be a substantial underestimate of the true overall neoplasia mortality as, in addition to the 12.43% of deaths ascribed directly to neoplasia, large components of the deaths ascribed to brain disorder (9.54%), mass lesion (8.38%) and death with an unrecorded cause (21.10%) may also have involved neoplasia. There were no differences detected between male and female for disorders as a cause of mortality, but white Boxers showed evidence of higher probability of death related to brain disorder and enteropathy than non-white Boxers. Despite the possibility of false positive findings in the current study, given the neurological component to the aetiological pathway for sensorineural deafness linked to white colour in dogs [71] and given the increased prevalence for brain disorder as a grouped disorder already reported in the current study, further work on an association between white colouration and neurological disorders is warranted.

This study had some limitations in addition to those previously reported for the application of veterinary primary-care clinical records as a research resource [19, 22]. The current study aimed to report the annual prevalence of common disorders in Boxers and therefore did not extract information about the date of first diagnosis, which would have allowed additional reporting of annual incidence. Additionally, information was not extracted on the severity or duration of these disorders, which would have been needed for a fuller evaluation of the welfare impact [58]. The current study reported results from multiple analyses so there are risks of false positive findings (type I error) as discussed above [76]. Consequently, the individual analyses in the current study should be considered as ‘hypothesis-generating’ rather than ‘hypothesis-confirmatory’ [77] and the results reported here should be considered in the light of other studies and incorporated within each of our individual understandings to optimise interpretations and applications.

## Conclusions

In conclusion, the current study identifies that the Boxer comprises around 1% of dogs in the UK, with around 10% of these Boxers being white. There was minimal evidence of noteworthy health differences between male and female Boxers, or between white and non-white Boxers. Among the four most common disorders recorded in Boxers, two are typically common across all types of dogs (otitis externa and periodontal disease) while two showed strong predisposition in the Boxer breed (epulis and corneal ulceration), suggesting the value of understanding breed-specific disorder patterns to offer insights for potential health interventions. The overall longevity of Boxer dogs was consistent with dogs of other breeds of similar body size.

## Data Availability

The datasets generated during and/or analysed during the current study will be made available at the RVC Research Online repository.

## Abbreviations

BAER: Brainstem auditory-evoked response
CI: Confidence interval
EPR: Electronic patient record
IQR: Interquartile range
KC: The Kennel Club
RVC: Royal Veterinary College
SD: Standard deviation
